# Niacin Enhancement for Parkinson’s Disease: An Effectiveness Trial

**DOI:** 10.3389/fnagi.2021.667032

**Published:** 2021-06-17

**Authors:** Raymond Chong, Chandramohan Wakade, Marissa Seamon, Banabihari Giri, John Morgan, Sharad Purohit

**Affiliations:** ^1^Department of Interdisciplinary Health Sciences, Augusta University, Augusta, GA, United States; ^2^Charlie Norwood Veterans Affairs Medical Center, Augusta, GA, United States; ^3^Department of Neuroscience and Regenerative Medicine, Augusta University, Augusta, GA, United States; ^4^Department of Neurology, Augusta University, Augusta, GA, United States; ^5^Center for Biotechnology and Genomic Medicine, Augusta University, Augusta, GA, United States; ^6^Department of Undergraduate Health Professions, Augusta University, Augusta, GA, United States

**Keywords:** B3, inflammation, nicotinic acid, UPDRS, fatigue, GPR109A, antiinflammation

## Abstract

We previously reported that individuals with Parkinson’s disease (PD) present with lower vitamin B3 levels compared to controls. It may be related to carbidopa interaction, defective tryptophan metabolism, and stresses of night sleep disorder. Vitamin B3 is the energy source for all cells by producing NAD^+^ and NADP^+^ in redox reactions of oxidative phosphorylation. Thus, some symptoms of PD such as fatigue, sleep dysfunction, and mood changes may be related to the deficiency of vitamin B3. Here, we conducted an effectiveness trial to determine the effect of 12 months of low-dose niacin (a vitamin B3 derivative) enhancement in PD individuals. An average of 9 ± 6-point improvement in the Unified Parkinson’s Disease Rating Scale (UPDRS) III (motor) score was observed after 12 months of daily niacin compared to the expected decline in score (effect size = 0.78, 95% CI = 7–11). Additionally, secondary outcome measures improved. Notably, handwriting size increased, fatigue perception decreased, mood improved, frontal beta rhythm during quiet stance increased, and stance postural sway amplitude and range of acceleration decreased. Set shifting, however, as measured by the Trail Making-B test, worsened from 66 to 96 s. Other measures did not change after 12 months, but it is not clear whether this represents a positive benefit of the vitamin. For example, while the quality of night sleep remained the same, there was a trend towards a decrease in the frequency of awakening episodes. These results suggest that niacin enhancement has the potential to maintain or improve quality of life in PD and slow disease progression.

## Introduction

Parkinson’s disease (PD) patients have lower than normal vitamin B3 levels compared to those of their spouses (Wakade et al., 2014, 2015). Vitamin B3 levels in the red blood cells as well as its metabolites in fasting plasma were lower by more than three standard errors (Wakade et al., 2014). NAD^+^ supplied by vitamin B3 is the energy source for cells by boosting mitochondrial functions (Wakade and Chong, 2014; Seamon et al., 2020). Some symptoms of PD such as fatigue, sleep dysfunction, and mood changes appear to be consistent with the deficiency of vitamin B3 (Wakade et al., 2014). This deficiency may be related to carbidopa interaction, defective tryptophan metabolism, and stresses of night sleep disorder (Wakade and Chong, 2014). The levodopa (L-DOPA) medication that is commonly used to treat PD symptoms depletes niacin levels by interfering with tryptophan breakdown. Intraperitoneal administration of L-DOPA (100 and 200 mg/kg) in rat brain decreases tryptophan, tyrosine, and serotonin to their lowest levels after an hour (Karobath et al., 1971). Tryptophan metabolism is also impaired in patients, as reported in individuals who were diagnosed with PD but had not yet been treated with anti-PD drugs (Ogawa et al., 1992). Niacin metabolites increase both tyrosine hydroxylase and dopamine levels *in vitro* by enhancing the recycling of quinonoid dihydrobiopterin to tetrahydrobiopterin (Vrecko et al., 1993, 1997).

Thus, there is growing evidence that PD patients need to overcome the low vitamin B3 levels through supplementation, more so than simply consuming B3-rich food (Hellenbrand et al., 1996). Here, we report the results of a 12-month effectiveness trial to determine the effect of low-dose over-the-counter niacin (a B3 derivative) enhancement in PD individuals on symptom and biochemical outcomes.

## Materials and Methods

### Subjects

Forty-seven subjects diagnosed with idiopathic PD participated in the study that was approved by the institutional review board. Diagnosis was based on the UK PD Society Brain Bank Diagnostic Criteria as well as the expert opinion of a neurologist (JM). There were 32 men and 15 women in the study. Their ages averaged 62 ± 5 years, disease severity was 2 ± 0.8 on the Hoehn and Yahr scale (H&Y; median = 2), duration of the disease was 6 ± 5 years (median = 5). All subjects were tested in the morning while in their On state. The mean interval between the last medication cycle and the beginning of testing was 2.5 ± 2.3 h. None of the subjects had dyskinesia or motor complications, which may confound their participation in the study. A summary of the subjects’ characteristics is presented in Table 1. Subjects were not withheld from taking their prescribed PD and/or other medications during the study.

**Table 1:** Parkinson’s disease (PD) characteristics.

	Placebo group	100-mg group	250-mg group	Overall mean	*p*
Age (years)	62 ± 4 (55–68)	64 ± 5 (55–76)	61 ± 6 (52–70)	62 ± 5 (52–76)	n.s.
Disease duration (years)	7 ± 6 (1–22)	5 ± 4 (1.5–15)	5 ± 3 (0.5–10)	6 ± 5 (1–22)	n.s.
UPDRS III (points)	21 ± 11 (7.5–39.5)	22 ± 14 (2.5–65.5)	22 ± 4 (6.5–55.5)	22 ± 13 (2.5–65.5)	n.s.
H&Y (median)	2 (1–3.5)	2 (0.5–4)	2 (0.5–3)	2 (0.5–4)	n.s.
MMSE (points)	29.8 ± 0.8 (29–30)	29.9 ± 0.3 (29–30)	29.8 ± 0.6 (29–30)	29.8 ± 0.6 (29–30)	n.s.
Carbidopa (mg/day)	115 ± 15 (0–200)	58 ± 23 (0–300)	95 ± 64 (0–200)	90 ± 75 (0–300)	n.s.
Levodopa (mg/day)	447 ± 59 (0–800)	270 ± 106 (0–1,200)	380 ± 67 (0–800)	367 ± 311 (0–1,200)	n.s.
Time between medication and test (hour)	2.3 ± 1.4 (0.5–6.0)	2.9 ± 3.6 (0.0–15.0)	2.2 ± 1.2 (0.5–4.0)	2.5 ± 2.3 (0.0–15.0)	n.s.

*UPDRS III, motor section of the Unified Parkinson’s Disease Rating Scale. H&Y, Hoehn and Yahr disease rating scale (Goetz et al., 2004). MMSE, Mini-Mental State Examination (Folstein et al., 1975). ± = standard deviation; ( ) = range; n.s., non-significant ANOVA test*.

#### 3-Month Double-Blinded Run-in Trial

**Figure 1:**
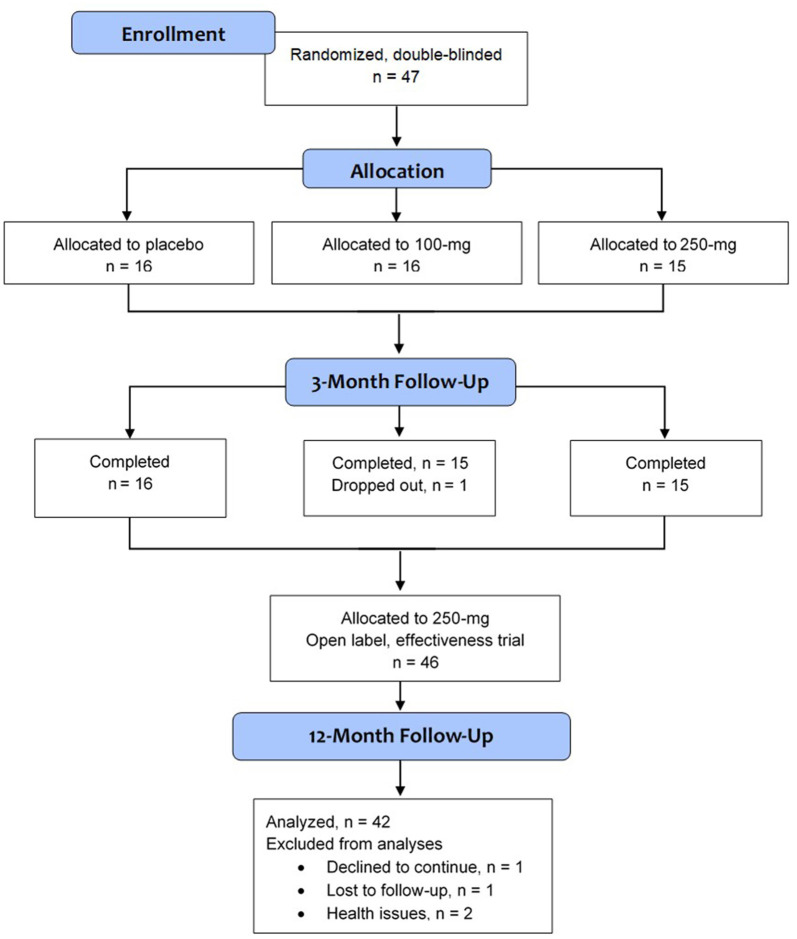
Flow diagram of the study design.

We started subjects on a double-blinded 3-month run-in dose-dependent supplementation to ensure that there were no adverse reactions. Subjects were randomly assigned to the placebo (*n* = 16), 100-mg (*n* = 15), and 250-mg (*n* = 15) slow-release niacin groups (Figure 1). To ensure that disease severity was comparable among the three groups, we used blocked randomization based on the H&Y disease severity (Group 1 = 1–2 and Group 2 = 2.5–4) to determine group assignments. Supplements were taken once daily.

Each subject was assessed at baseline and 3 months after with the following:

(1) Clinical tests

(a) Motor Function Measures

(i) Unified Parkinson’s Disease Rating Scale (UPDRS) III (Motor section; as the primary outcome measure)
(ii) Handwriting size
(iii) Pentagon drawing size (both from the Mini-Mental State Examination; Folstein et al., 1975)
(iv) Quiet 30-s stance (APDM system; Horak and Mancini, 2013)

(b) Non-Motor and Quality of Life Measures

(i) Fatigue Severity Scale (Krupp et al., 1989)
(ii) Fatigue Visual Analog Scale (Chong et al., 2018)
(iii) Geriatric Depression Scale (GDS-32; Yesavage et al., 1982)
(iv) Parkinson’s Disease Questionnaire (PDQ-8; Jenkinson et al., 1997) for quality of life
(v) PD sleep quality questionnaire (Chaudhuri et al., 2002)
(vi) Postural instability questionnaire (Chong et al., 2011, 2012)
(vii) Stroop test (word–color conflict component; Dujardin et al., 1999)
(viii) Trail Making Test (Reitan and Wolfson, 1993; Misdraji and Gass, 2010)
(ix) Electroencephalogram (EEG) recordings during night sleep (Zeo system; Griessenberger et al., 2013; Wakade et al., 2014; Cellini et al., 2015)
(x) Frontal (FP1) beta rhythms (13–30 Hz) during the physical function tests (MindWave system; Rieiro et al., 2019)

(2) Biochemical test

Venous blood sample was collected from all the subjects at the end of the clinical tests. Plasma was separated by centrifugation at 1,800 *g* for 10 min, divided into 1-ml aliquots, and stored at −80°C until use. Peripheral blood mononuclear cells (PBMCs) were collected from the buffy coat, further cleared of the red blood cells, and stored pelleted at −80°C until use. Standard protocols for western blot, high-performance liquid chromatography mass spectrometry (HPLC/MS), and chromatogram analyses (Wakade et al., 2014, 2018; Giri et al., 2019) were carried out to determine changes in niacin plasma levels and expression of the GPR109A protein receptor, in which niacin binds with high affinity (Wakade et al., 2014; Seamon et al., 2020). In addition, we analyzed 12 cytokine levels to explore whether these inflammatory markers may also change with niacin supplementation (IL-1β, IL-2, IL-6, IL-8, IL-10, IP-10, IFN-γ, MCP-4, MIP-1α, MIP-1β, TNF-α, and SAA). Details of the biochemical analyses are provided in the Supplementary Material.

Eighty percent of the subjects in the 100-mg group reported experiencing flushing during this period (from occasional to frequent), which is a common feature of niacin supplementation. Within the 250-mg group, 5% of subjects experienced the flushing response. In all cases, the symptom lasted 30–45 min, after which no other symptoms nor events occurred.

#### 12-Month Open-Label Effectiveness Trial

Following the 3-month test, a 12-month effectiveness trial (Gartlehner et al., 2006) of fixed-dose 250-mg slow-release daily niacin was carried out. Subjects in the placebo and 100-mg groups switched to the 250-mg regimen for 12 months, while the 250-mg group continued the same dosing regimen for 9 months (to also achieve 12 months of 250-mg supplementation). The same clinical and biochemical assessments were administered to the subjects at the end of the study.

### Statistics

The primary clinical outcome was the UPDRS III (motor section) score. We used the primary endpoint to calculate the required sample size. We invoked rejection of the null hypothesis when the mean score at 12 months is less than that at baseline. The margin of superiority that represents the minimal clinically meaningful change in score, *δ*, is 5 (based on the pilot data of 15 subjects). This value is consistent with the expected median annual rate of decline in the UPDRS III score of +5.5 points (Poewe, 2006; Evans et al., 2011).

For a significance level (*α*) of 5%, a power (1 − *β*) of 80%, and an expected standard deviation of the difference between pairs of data at 3.9, the minimum sample size was nine participants (GraphPad Statmate 2.0, San Diego, CA, USA). Our study sample was substantially increased to take into account potential dropouts, any unexpected increase in variability, and the use of multivariate statistical analyses. Intention-to-treat (ITT) analysis to account for dropouts were carried out with the last-observation-carried-forward method by using the 3 months post data in place of missing values.

Each clinical outcome measure within each category was analyzed with Hotelling’s *T*-square multivariate analyses on the pre-niacin and 12-month post-niacin scores to test the null hypothesis that the population vectors are the same; i.e., posttreatment scores are not different from those of the baseline. Simple effect analyses (paired *t*-tests) were done to determine if they corroborated the multivariate test results.

Blood samples were analyzed with provided information about changes in GPR109A receptor and niacin levels, before and after niacin supplementation. To determine the effect of 12-month post-niacin intervention on the blood markers, 3 (group) × 3 (period) mixed ANOVAs were conducted. A significant interaction or main effect was followed by simple effect analyses (paired *t*-tests). Cytokines were analyzed with simple effect tests.

Alpha was set at 0.05 for all analyses.

## Results

Of the 47 subjects who enrolled in the study, 46 completed the 3-month trial. Of these, 42 finished the 12-month trial (Figure 1). One subject declined to continue, one was lost to follow-up, and two subjects were unable to complete the duration due to worsening comorbidities that were unrelated to PD.

## Primary Outcome: UPDRS III

### Per-Protocol Analysis

As can be seen in Table 2, a decreased average score of 3.5 ± 6 points (indicating an improvement) in the UPDRS III score was observed after 12 months of daily niacin, from 21.5 ± 12.9 to 17.7 ± 11.7 points, *t*_(41)_ = 3.6, *p* = 0.0009, for a two-sided test. The reversal in symptoms observed in this study represents an average improvement of 9 ± 6 points (−16%) in the UPDRS III based on the expected 5.5-point decline minus the observed −3.5-point improvement, effect size (ES) = 0.78, 95% confidence interval (CI) = 7–11, which translated to 99% power of detection for a two-sided paired test. UPDRS scores at 3 months were variable as expected, since niacin is not a drug. Only the 100-mg group was detected to have improved with the supplementation, *p* = 0.0076.

**Table 2:** Clinical outcome measures.

	Mean at baseline (SD)			Mean at 3 months (SD)			Mean at 12-months (SD)	Change (Baseline vs. 12 months)	Effect size*	Univariate test*	Multivariate test*
	*p*	100 mg	250 mg	*p*	100 mg	250 mg				*p*-value	
**Primary outcome: UPDRS III (motor)**, points	20.9 (10.9)	22.1 (14.3)	21.6 (14.3)	19.0 (12.0)	17.6 (13.2)	22.6 (18.7)	17.7 (11.7)	−17.3%	0.32	<0.0006	NA
**Physical Functioning**
Sentence height (cm)	0.9 (0.2)	0.8 (0.3)	0.9 (0.3)	1.0 (0.03)	1.0 (0.4)	1.0 (0.4)	1.2 (0.4)	33%	0.91	<0.0001	1.2
Sentence area (cm^2^)	7.6 (2.6)	6.0 (2.3)	7.1 (3.2)	8.5 (3.1)	8.2 (5.2)	8.0 (4.4)	9.9 (4.2)	38%	0.76	<0.0001	−0.83
**Cognitive Functioning**
Fatigue severity scale (points)	41.2 (10.9)	43.2 (10.4)	44.7 (12.5)				31.4 (8.6)	−26%	1.17	<0.0001	0.93
Geriatric depression scale (points)	7.6 (6.6)	14.1 (9.2)	13.1 (9.7)		Not assessed		4.8 (5.5)	−58%	0.92	0.0005	0.70
Fatigue visual analog scale (number)	6.5 (2.2)	4.3 (6.8)	4.6 (6.6)				7.1 (1.9)	35%	0.88	0.0002	0.27
Trail making B first trial (s)	73.6 (30.5)	109.3 (138.6)	95.3 (70.8)	69.7 (44.9)	104.7 (123.5)	92.5 (72.8)	95.6 (63.3)	45%	0.66	0.043	0.93
Sleep questionnaire (points)	107.4 (24.0)	111.1 (18.6)	105.4 (21.2)	109.1 (24.5)	115.4 (18.9)	107.1 (21.4)	117 (22)	7.5%	0.36	0.028	1.03
**Frontal Beta Rhythm**
30-s quiet stance** (%)	55.2 (16.6)	54.6 (17.0)	58.1 (16.5)	52.4 (19.0)	59.2 (19.1)	47.4 (18.4)	60.9 (13.6)	13%	0.43	0.066	0.41
**Stance Postural Stability**
Centroid frequency ML (Hz)	1.3 (0.5)	1.3 (0.2)	1.3 (0.5)	1.3 (0.4)	1.1 (0.4)	1.3 (0.4)	1.09 (0.3)	−15%	0.54	0.009	0.78
Median frequency ML (Hz)	0.9 (0.6)	1.0 (0.4)	0.8 (0.5)	1.0 (0.6)	0.8 (0.4)	1.0 (0.6)	0.7 (0.5)	−25%	0.49	0.023	0.06
95% circle sway area (m^2^/s^4^)	0.1 (0.03)	0.2 (0.5)	0.1 (0.1)	0.1 (0.1)	0.1 (0.1)	0.1 (0.1)	0.05 (0.03)	−36%	0.46	0.034	−1.40
Frequency dispersion ML AD	0.7 (0.1)	0.6 (0.1)	0.7 (0.1)	0.6 (0.1)	0.7 (0.1)	0.6 (0.1)	0.7 (0.1)	7%	0.45	0.014	−0.20
95% frequency ML (Hz)	2.8 (0.5)	2.8 (0.3)	2.7 (0.6)	2.7 (0.5)	2.5 (0.6)	2.6 (0.6)	2.5 (0.6)	−8%	0.41	0.031	0.29

The primary outcome variable (UPDRS III) and other secondary measures in the table indicate an improvement in scores following 12 months of daily 250-mg niacin enhancement. The exception is the first trial of the Trail Making Test, which worsens with disease progression in spite of the niacin supplementation. Other variables were unchanged and are not shown in the table. P, placebo group, 100 mg, 100-mg group, 250 mg, 250-mg group. *The univariate test is based on the one-tailed paired *t*-test (baseline vs. 12 months). The multivariate test is based on Hotelling’s *T*-square canonical discrimination pooled within-class standardized canonical coefficients (baseline vs. 12 months). Effect size is based on Cohen’s *d* (baseline vs. 12 months). **Raw signals were amplified, while ambient noise was removed. The device’s proprietary algorithm was then applied to determine the subjects’ mental states. Higher values indicate an improvement in score. It may mean less distraction, less wandering thoughts, better focus, or decreased anxiety. ML, mediolateral plane.

### Intention-to-Treat Analysis

Inclusion of the four subjects who dropped out of the 12-month intervention produced the same results: UPDRS III improved from 21.5 ± 12.9 to 18.1 ± 13.0 points, *t*_(45)_ = 3.9, *p* = 0.0003, representing an overall 9 ± 6-point swing in score (ES = 0.68, 95% CI = 7–11 at 99% power of detection).

There was no association between the UPDRS score at baseline or 12 months and disease severity, duration of disease, or carbidopa intake.

### Secondary Outcomes

#### Clinical Measures

Many secondary outcome measures also improved. Particularly, handwriting size increased, perception of fatigue decreased, mood improved, frontal beta rhythm during quiet stance increased, and stance postural control improved. Set shifting as inferred from the Trail Making Test worsened from 66 to 96 s.

Other measures did not change after 12 months, but it is not clear whether this represents a positive benefit of the vitamin. For example, while the quality of night sleep remained the same at 79% efficiency ± 14 and 15, respectively (*p* = 0.47), there was a weak trend and small effect towards a 10% decrease in the average frequency of awakening episodes, from 8 to 7 times nightly, ES = 0.23, *p* = 0.18.

#### Biochemical Measures

##### Niacin Plasma Levels

**Figure 2:**
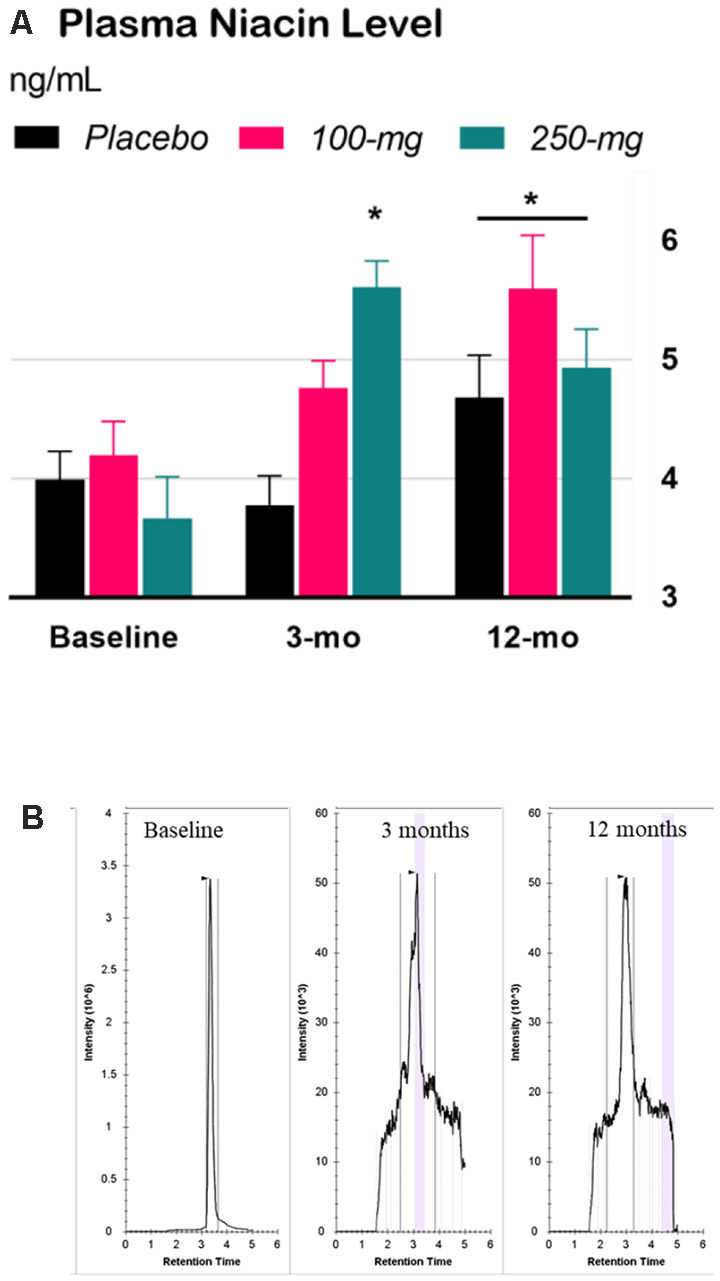
Effect of niacin on plasma levels. **(A)** High performance liquid chromatography (HPLC) analysis of niacin levels in plasma. Error bars are SEM. *Baseline*: niacin levels before treatment; *3-months* (double-blind phase): niacin levels after 3 months of treatment (placebo, 100-mg, or 250-mg daily niacin); *12-months* (effectiveness phase): niacin levels after all groups received 12 months of 250-mg dailyniacin. *p < 0.05 compared to baseline. **(B)** Chromatograms were acquired for baseline, 3 months, and 12 months based on the retention time in size exclusion chromatography.

The interaction effect in the two-way Time × Group mixed ANOVA was significant, *F*_(4,65)_ = 4.84, *p* = 0.0018. Tukey’s simple effect analyses on each group showed that niacin levels decreased at 3 months for the 250-mg group only (*p* < 0.0001). At 12 months, niacin levels increased across the groups by an average of 34% compared to those at baseline, *p* < 0.0001, ES = 1.2 (Figure 2).

##### GPR109A Protein Expression

**Figure 3:**
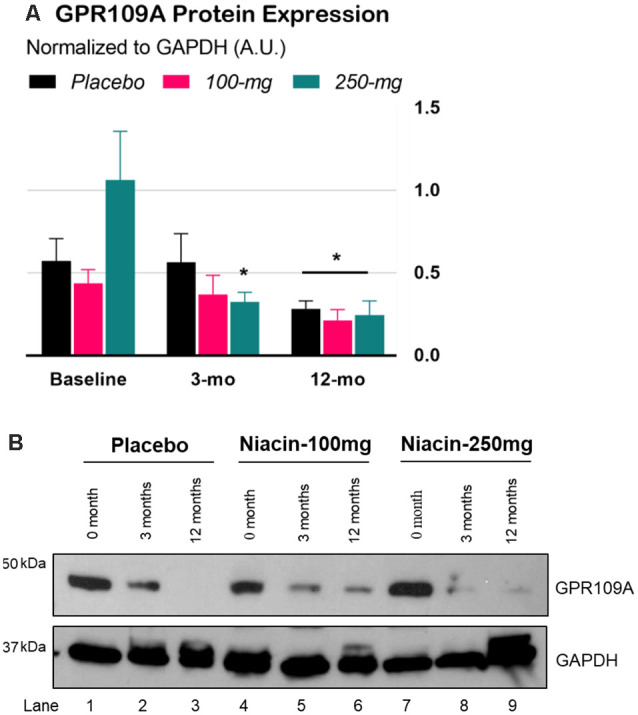
Expression of GPR109A protein in peripheral blood mononuclear cells (PBMCs) of Parkinson’s disease (PD) patients. **(A)** Densitometry analysis of GPR109A proteins normalized with GAPDH protein. Error bars are SEM. *Baseline*: GPR109A levels before treatment; *3-mo* (double-blind phase): GPR109A levels after 3 months of treatment (placebo, 100-mg, or 250-mg daily niacin); *12-mo* (effectiveness phase): GPR109A levels after all groups received 12 months of 250-mg daily niacin. *p < 0.05 compared to baseline. **(B)** Expression of GPR109A measured by immunoblot in isolated white blood cells from PD subjects treated with either placebo or niacin at two dosages for the indicated time points (upper panel). GAPDH is used for equal loading control of total protein on SDS-PAGE gels (lower panel).

The interaction effect in the two-way Time × Group mixed ANOVA was significant, *F*_(4,72)_ = 3.06, *p* = 0.022. Tukey’s simple effect analyses on each group showed that GPR109A levels decreased in the 250-mg group at 3 months, *p* = 0.0002. At 12 months, GPR109A levels across the groups decreased by an average of 172% compared to those at baseline, *p* = 0.0002, ES = 0.84 (Figure 3).

There was no association between the UPDRS score at baseline or 12 months and niacin or GPR109A levels.

##### Cytokines

Three out of the 12 cytokines increased following 12 months of niacin supplementation: (1) IL-10, from 0.33 ± 0.17 to 0.41 ± 0.20 pg/ml, ES = 0.45, *p* = 0.012; (2) MIP-1α, from 16.27 ± 5.12 to 18.99 ± 7.85 pg/ml, ES = 0.42, *p* = 0.03; and (3) IL-1β, from 0.04 ± 0.03 to 0.08 ± 0.04 pg/ml, ES = 1.1, *p* = 0.004. The remaining nine cytokines did not change. The magnitude of increase in the three cytokines did not correlate with the corresponding change in niacin nor GPR109A levels. There was a moderate correlation between the change in MIP-1 α and IL-10 (*r* = 0.44, *p* = 0.005) as well as IL-1β (*r* = 0.47, *p* = 0.055).

## Discussion

Considering that PD is a progressive condition, the observed improvement in the UPDRS III score following 12 months of niacin daily supplementation is substantial and clinically meaningful. Other potentially promising outcomes which were observed in hand drawing size, postural stability, frontal EEG rhythm, fatigue, and mood are interesting and warrant further investigation to study their mechanisms. For example, the improvement in frontal beta rhythm during standing could mean several things. It may mean less distraction, less wandering thoughts, better focus, or decreased anxiety.

The improvement in stance postural control was found to occur primarily along the mediolateral plane at a magnitude of 0.47 effect size, which is clinically meaningful as it indicates that subjects could perceive the improvement. Mediolateral postural control during stance and walking is indicative of balance control ability (as opposed to anteroposterior control; Mancini et al., 2012). The improvement may reflect a combination of factors such as decreased fatigue, more efficient integration of sensorimotor processing, better mood, and increased wakefulness, all of which were found to improve to some extent in this study.

It is interesting to note that cognitive set shifting deficits, a hallmark of PD decline (Cools et al., 1984; Hayes et al., 1998; Chong et al., 1999, 2000), did not improve with niacin as inferred from the Trail Making Test. Cognitive set shifting is complex. It invokes executive control involving the frontal regions. The explanation(s) for the worsening performance in this test is equally complicated and may involve nondopaminergic and other neural mechanisms that appear to be unresponsive to niacin intervention. Perhaps a longer duration of supplementation is needed to observe an arrest or reversal in the decline of performance. Nevertheless, the other reported measures of improvements are intriguing, in that they contribute to the overall improvement in quality of life (Chong et al., 2018).

Similarly interesting are other outcome measures which had little if any change after 12 months of niacin, because a lack of worsening symptoms may represent a positive benefit of the supplementation. For example, while the quality of night sleep appears to have improved, there was also a weak trend towards a decrease in the frequency of awakening episodes. Because frequent awakenings are detrimental to sleep quality and daytime functioning, the lack of deterioration may represent a positive benefit of the supplementation.

The anti-inflammatory portion of the niacin mechanism is mediated though its receptor GPR109A. The corresponding decrease in GPR109A levels is consistent with our previous finding of a change in macrophage polarization from M1 (pro-inflammatory) to M2 (counter-inflammatory) profile acting through the niacin receptor GPR109A along with an improvement in quality of life (Wakade et al., 2018). Niacin reduces the nuclear translocation of phosphorylated nuclear factor kappa B (p-NF-κB) induced by lipopolysaccharide in the nucleus of RAW264.7 cells. This in turn decreases the expression of pro-inflammatory cytokines including IL-1β and IL-6 in RAW264.7 cells. This decrease in the nuclear translocation of p-NF-κB and the expression of pro-inflammatory cytokines is not observed after GPR109A knockdown in RAW264.7 cells (Giri et al., 2019).

The implication of a decrease in inflammation as measured by the GPR109A following niacin supplementation needs further investigation (Seamon et al., 2020) including how it impacts the immune system and its influence on cytokine levels. For example, while the anti-inflammatory IL-10 cytokine also increased, pro-inflammatory cytokines IL-1β and MIP-1α increased as well. Increased IL-10 demonstrates enhanced neuroprotection. A recent PD study demonstrates lower MIP-1α levels in PD subjects compared to healthy controls. The increase in MIP-1α observed in our study may indicate improved repair ability in PD subjects (Calvani et al., 2020) and may reflect on macrophage polarization modulation with niacin intervention. Impairment of cytokine production in plasma is noted in PD (Hasegawa et al., 2000). Therefore, a temporary increase of IL-1β after niacin intervention may demonstrate normalization of the immune response. An accurate assessment of the immune response is possible with more frequent plasma sample analysis for cytokines instead of a “snapshot” approach.

## Conclusion

We have demonstrated the potential effectiveness of over-the-counter niacin enhancement as a proof of concept to support the well-being of individuals with PD. Vitamin B3 augmentation has the potential to maintain or improve symptoms. Based on the results of this effectiveness exploratory trial, a larger multicenter RCT is warranted.

## Data Availability Statement

The raw data supporting the conclusions of this article will be made available by the authors, without undue reservation.

## Ethics Statement

The studies involving human participants were reviewed and approved by Augusta University. The patients/participants provided their written informed consent to participate in this study.

## Author Contributions

RC and CW: conceptualization, visualization, and supervision. RC, CW, BG, SP, and MS: methodology, validation, investigation, resources, and data curation. RC, CW, and JM: writing—original draft preparation. RC, CW, BG, SP, MS, and JM: writing—review and editing. RC: formal analysis, project administration, and funding acquisition. All authors contributed to the article and approved the submitted version.

## Conflict of Interest

The authors declare that the research was conducted in the absence of any commercial or financial relationships that could be construed as a potential conflict of interest.
