# Supplementary material for: Niacin Enhancement for Parkinson’s Disease: An Effectiveness Trial
Source: Front Aging Neurosci. 2021 Jun 17;13:667032. doi: 10.3389/fnagi.2021.667032 (PMC8245760; doi:10.3389/fnagi.2021.667032)
Supplement: Supplementary file 1 [file Data_Sheet_1.pdf]

Supplementary section

Biochemical Analyses

a) Quantitation of Niacin Plasma

The quantitative analysis for niacin was performed at the core facility lab, Augusta University on a HPLC (Shimadzu, Torrance, CA) coupled to using LC-MS/MS, Liquid Chromatography with tandem mass spectrometer detector (Thermo,Waltham, MA). Briefly, 0.10 mL aliquot of plasma samples were mixed with 0.4 mL of methanol and then vortexed for 3 min followed by incubation of 10 min at room temperature. Samples were centrifuged at 15000g for 5 min at room temperature. 450µl of supernatant was transferred to a new tube and subjected to vacuum centrifugation until dry. The residue was reconstituted in 45µl of 2% acetonitrile in 0.1% formic acid. Reconstituted samples were separated on C18 column (100x2.1mm, 1.7um, Phenomenex, Torrance, CA) and eluted using 2% acetonitrile with 0.1% formic acid at a flow rate of 0.2ml/min and a column temperature of 40°C. The eluant was electro-sprayed into the MS via electrospray ionization in positive mode. The MS based identification was done in SRM/MRM mode with the following instrument settings: ion spray voltage 3500V, sheath gas 35, aux gas 10, ion transfer tube temperature 325, vaporizer temperature 250, and Q1 and Q3 resolution 0.7 FWHM. The optimal fragments (Q3), collision energy (V) and RF lens (V) were determined using commercial niacin standards.

b) GPR109A Expression Levels in PBMC's

Equal amounts of protein from PBMC's were loaded into 4%–15% BIORAD SDS–PAGE gels and ran at 120 volts followed by transfer of protein to PVDF membrane as described in the Giri

et al. 2019. Briefly, membranes were incubated with 5% non-fat dry milk (NFDM) for one hour at room temperature. After blocking, membranes were incubated with a GPR109A primary antibody at 1:1000 dilution (Bioworld Technology, Inc. Cat. No.# BS2605) overnight at 4ºC. Membranes were washed three times with phosphate-buffered saline with 0.05% tween 20 (PBS-T) for 10 min with gentle agitations. Blots were incubated with the secondary antibody (1:5000 dilutions) for one hour at room temperature, followed by washing (3 x10 min) with PBS-T. After incubation, followed by washing, the membranes were developed using peroxidase-conjugated anti-mouse IgG or anti-rabbit IgG antibodies and subsequently developed using Denville Scientific Inc. ECL kit (Swedesboro, NJ, USA). For GAPDH, membranes were subsequently reprobed with a mouse monoclonal antibody against GAPDH (Sigma, San Diego, Cat. No. G9295). The analyses of band intensity were performed using Image J software.

c) Plasma Levels of Inflammatory Markers

We measured levels of six cytokines (IL-1β, IL-2, IL-6, IL-8, IL-10, IFN-γ and TNF-α), four chemokines (IP-10, MCP-4, MIP-1α and MIP-1b) and acute phase protein (SAA) in serum of all the study subjects. Serum levels were measured using electro-chemiluminiscence using Meso Scale Discovery platform (Meso Scale Discovery, Rockville, MD). These analytes were selected based on their neuro-inflammmatory activity. The assay was performed according to the protocol provided by the manufacturer. Briefly serum samples (25µl) or calibrators (25µl) were added to antibody coated wells of 96 well plates and incubated for 2h with shaking. Samples were aspirated and washed three times with phosphate buffer containing 0.1% Tween-20 (PBS-T). The captured immunocomplexes were detected by Sulfo-Tag secondary antibody, the plate was incubated for 2h with shaking. The plates were washed again with three times with PBS-T. The

signal was captured by adding 150µl of read buffer, in a MESO Quickplex SQ 120 reader. To determine the concentration, the light intensity was regressed against the known standard using Discovery Workbench Software (v4.0, Meso Scale Discovery).
